# Age-related differences across the adult lifespan: a comparison of six field assessments of physical function

**DOI:** 10.1007/s40520-025-02965-1

**Published:** 2025-03-08

**Authors:** Lien Meulemans, Jolien Deboutte, Jan Seghers, Christophe Delecluse, Evelien Van Roie

**Affiliations:** 1 Department of Movement Sciences, Physical Activity, Sports & Health Research Group, KU Leuven, Tervuursevest 101 - bus 1501, Leuven, 3001 Belgium; 2 Faculty of Rehabilitation Sciences, University of Hasselt, REVAL-Rehabilitation Research Center, Wetenschapspark 7, Diepenbeek, 3590 Belgium

**Keywords:** Stair climbing, Muscle power, Ageing, Inertial measurement unit, Physical capacity, Functional performance

## Abstract

**Background:**

Age-related declines in physical capabilities often result from decreased lower-limb muscle strength and power, which are measurable through field tests. Various tests can detect functional declines in older adults, but their responsiveness to age-related differences is less understood in those without substantial impairments. Therefore, this study evaluated and compared the ability of field tests to detect age-related changes in physical and muscle function across adulthood.

**Methods:**

304 participants (52% female; 19–85 years) completed six field tests: handgrip strength (HGS), maximal gait speed (MGS) over a 10-m course, 5-repetition sit-to-stand power (STSP), timed up and go (TUG), countermovement jump (CMJ), and stair climbing power (SCP). Segmented regression analysis determined the relationship between age and field test performance, and identified the age at which the rate of decline increased. A multilevel linear mixed model compared decline rates between tests.

**Results:**

Before 60 years, SCP and CMJ were responsive to age-related differences (-0.70 to -0.81%/year, *p* < 0.05), whereas TUG and STSP (lower age-related decline, -0.18% to -0.52%/year, *p* < 0.05) and HGS and MGS (no significant age-related decline) exhibited lower responsiveness. After 60, most tests (except the STSP) demonstrated increased responsiveness to age-related differences, although these differences remain most pronounced in SCP and CMJ (-1.61 to -1.75%/year, *p* < 0.05).

**Conclusions:**

These findings imply that most field tests are responsive to age-related declines in physical and/or muscle function after 60. In younger age groups, field tests that evaluate lower-limb power and have minimal ceiling effects, such as SCP and CMJ, should be prioritized.

## Introduction

The global increase in the older population has made the promotion of healthy ageing a worldwide priority [1]. A key aspect of healthy ageing is the maintenance of an individual’s physical capabilities that allow independent functioning [2]. Therefore, detecting early changes in an individual’s physical capabilities to enable targeted interventions is essential for ensuring that the additional years lived are characterized by good health.

Lower-extremity functioning plays a crucial role in maintaining independence among older adults [3]. Multiple, clinically feasible field tests have been developed to assess lower-extremity physical functioning in older adults, such as the maximal gait speed (MGS) [4, 5] and timed up and go (TUG) [6, 7] tests. While these tests can predict negative health outcomes in older adults, such as hospitalization-associated functional decline [8], disability [9], frailty status [10, 11], fractures [9] and mortality [12], the test procedures may not fully challenge individuals at the higher end of the performance spectrum who are still capable of running instead of walking. This may hamper the ability of the test to detect early changes in performance in robust, well-functioning middle-aged and older adults.

An important determinant of lower-extremity physical function in older age, is the capacity to produce high muscle strength and power levels [13]. Longitudinal research demonstrated that maximal muscle strength of the knee-extensor muscles declines by 0.7-1.0% per year in middle-aged adults (40–60 years) and by 1.1–1.3% in older adults (≥ 60 years) [14]. Maximal muscle power declines even more rapidly, with yearly decreases of 1.1–1.4% in middle age and 2.2–2.4% in older age, demonstrating a progressively blunted ability to produce force at moderate-to-high velocities in older age [14]. The greater decrement in force production during fast contractions can be explained by a combination of age-related changes at the single muscle fiber level, in the muscle-tendon architecture and in the neural drive to the muscle. For example, ageing is accompanied by a preferential loss of type II muscle fibers [15], a fast-to-slow shift in the single muscle fiber phenotype [16], reductions in tendon stiffness [17] and muscle fascicle shortening velocity [18], and impaired muscle gearing [19, 20]. Also, decreased neural drive during the early phase of muscle contraction [21] combined with increased coactivation of antagonist muscles [22] contribute to the age-related decline in maximal muscle power. Altogether, this evidence underscores the importance of assessing a person’s ability to exert high levels of power if the aim is to detect early signs of functional deterioration. The clinical relevance of lower-limb muscle power assessments is further supported by the association between reduced muscle power in old age and a variety of negative health outcomes, such as mobility limitations and disability [23, 24], cognitive decline [25], recurrent falls and fractures [26], and increased risk for hospitalization and mortality [27].

Assessments of muscle function can be conducted using various devices, such as isokinetic dynamometry or pneumatic leg press machines [28], but this equipment is expensive and challenging to implement in clinical or home-based settings. Field tests offer a practical alternative, being time-efficient, cost-effective, easy to administer, and requiring minimal equipment [29]. A simple and reliable measurement of muscle strength that is commonly used in clinical settings, is the handgrip strength (HGS) test. The HGS test reflects overall muscle strength and can predict muscle mass, independence of daily living and quality of life [30]. However, considering that muscle mass [31] and strength [32] of the upper limbs typically decline later compared to the lower limbs, the HGS test may not be sufficiently suited to detect early (i.e., from 40 years onwards) declines in physical capability. In addition, current methodologies used in clinical settings fail to provide information on rapid force production or dynamic (fast) contractions, which typically demonstrate more pronounced age-related declines [14, 33]. Therefore, it is recommended to supplement grip strength assessments with other measures, especially those that focus on lower-limb muscle power [34].

Considering the rapid technological advancements, wearable sensors present a promising approach for estimating lower-limb power output during functional activities, such as the 5-repetition sit-to-stand (STS) [35–37] and stair-climbing (SC) [36, 38–41] test. While the 5-repetition STS test is safe and reliable among older adults [42, 43], it may still be questioned whether the test procedure allows well-functioning adults to exert maximal levels of power production in the knee- and hip-extensor muscles during the concentric phase [44]. The test instructions prohibit jumping of the chair, which results in more ‘controlled’ movements. The SC test, in which vertical power production is estimated through a wearable sensor on the lower back, might be a valuable alternative to assess power production in well-functioning adults. The test appears to be highly related to leg press power [36] in adults aged 20–70 years, shows good reliability [39] and is able to detect age-related differences [38].

Another commonly used field measurement indicative of lower-limb muscle power, is the countermovement jump (CMJ). Previous research has demonstrated the ability of the CMJ to detect early deteriorations in muscle function and functional capacity [45]. In addition, CMJ performance is related to other tests assessing muscle function and functional capacity in older women (≥ 60 years), such as the short physical performance battery, gait speed, 5-repetition and 30-s STS, TUG, HGS and isokinetic knee-extension strength tests [46], making it a suitable candidate for preventative screening of lower-limb functioning.

In summary, various field tests are available to assess physical functioning in older adults. Performance on these tests is influenced by several physical attributes, including age-related changes in body composition [47], muscle architecture and metabolism [48], joint range of motion [49], the central and peripheral nervous system [50], and postural control [51]. While these tests have consistently shown age-related declines in performance throughout the adult life course [36, 38, 45, 52–56], a novel aspect of the current cross-sectional study is its focus on lower-limb muscle power, assessed through field tests such as STSP, CMJ, and SCP. These tests are compared to commonly used measurements of physical performance (MGS, TUG) and muscle strength (HGS). We hypothesized that CMJ and SCP, which require significant lower-limb muscle power production, would be more prone to age-related differences from as early as middle age onwards [14, 57], compared to tests that do not fully challenge individuals at a higher end of the performance spectrum (STSP, MGS, TUG) or that focus on upper-limb rather than lower-limb functioning (HGS) [32].

## Materials and methods

### Design and participants

This cross-sectional study was conducted at KU Leuven, approved by the Ethics Committee Research UZ/KU Leuven (S62540) in accordance with the Declaration of Helsinki, and registered as clinical trial on clinicaltrials.gov (NCT04019132). To ensure that participants would be recruited across the full adult lifespan, a target for sample size was set for four age groups: 19–39, 40–54, 55–64 and ≥ 65 years old, i.e., *N* = 25 per sex in the two youngest groups and *N* = 50 per sex in the two oldest groups, resulting in *N* = 300 participants for the analyses. The doubling of the number of participants in the older age groups was done because higher variability in performance was expected in those groups. Sample size calculation was performed in G*power version 3.1 to ensure that our targeted sample size would be sufficient to achieve appropriate statistical power. More specifically, to detect a medium effect size (f² = 0.15) in linear multiple regression analyses (fixed model), *N* = 138 should be sufficient to achieve statistical power of ≥ 0.95 (alpha 0.05, 5 predictors, see statistical analyses section), so our targeted sample size of *N* = 300 (*N* = 150 per sex) would suffice. This sample size would also be sufficient to detect a large effect size (power ≥ 0.80) for cross-level interactions (i.e., age-by-test, see statistical analyses section) based on the work of Arend and Schäfer (2019) [58].

All participants were recruited through local advertisements. Exclusion criteria were musculoskeletal injury, recent surgery, unstable cardiovascular diseases, inability to walk independently, dementia, acute infection and/or fever. A total of 324 participants (162 per sex) were assessed. However, due to technical errors, data were missing for at least one field test in 20 participants, resulting in a final sample of 304 participants (145 men, 159 women; age 19–86 years). All participants provided written informed consent before participation in the study.

### Anthropometric measurements

Height (m) was measured to the nearest millimeter using a portable stadiometer (Seca GmbH, Hamburg, Germany) and weight (kg) to the nearest 0.1 kg using digital scale (Seca GmbH, Hamburg, Germany). The measurements were used to compute body mass index (BMI; kg/m^2^).

### Field tests

All participants performed six field tests. Before each test, participants completed a sub-maximal practice trial. The test included 2–3 trials, with recovery time (1–2 min) between each trial, and the best trial was used for further analysis. The field test showed good to excellent reliability in our lab (in a sample of *N* = 32–56 community-dwelling older adults ≥ 65 years), with intraclass correlation coefficients (ICC’s) ranging from 0.75 to 0.95 and coefficients of variation (CV%) below 10%. ICC’s and CV% have been added below for each test.

#### Maximal gait speed (MGS)

Participants were instructed to walk a 10 m distance as quickly as possible. Timing gates (Racetime2 Light Radio, Microgate, IT) recorded the time taken (s), which was converted to m/s for easier comparison with other results. ICC = 0.75, CV% = 5.88.

*Timed-up-and-go (TUG).* Participants were instructed to complete the test as fast as possible. They stood up from a a standardized chair (0.46 m height, no armrests), walked to a line on the floor placed 3 m away, turned around behind the line, returned to the chair and sat down. A stopwatch was used to record the time (s) needed to perform the test [59]. ICC = 0.78, CV% = 5.12.

#### Handgrip strength (HGS)

Grip strength (kg) of the dominant hand was measured using the Jamar^®^ handgrip dynamometer, which was adjusted for hand size. Participants were instructed to sit with a straight back, feet flat on the ground and their non-dominant arm resting on their legs. The elbow of the dominant arm was flexed at a 90° angle, with the upper arm positioned vertically and the forearm in a horizontal, neutral position. Participants were instructed to exert their maximum grip strength [60]. ICC = 0.95, CV% = 8.07.

#### 5 Times sit-to-stand (STS) power test

The STS test was performed on a standardized chair (0.46 m height, no armrests). Participants performed five full cycles of STS movements as rapidly as possible with arms crossed over the chest. Full seating position was defined as back touching the backrest, and full standing position as standing straight. Participants wore a sensor (DynaPort MoveTest, McRoberts, The Hague, NL) positioned at the lumber spine and secured with an elastic belt. Mean STS power was determined from the sensor data for each transition phase from sit to stand following established procedures [36]. The average power over the five STS cycles was computed (STSP; W) and used in further analyses. ICC = 0.93, CV% = 7.23.

#### Countermovement jump (CMJ)

Participants were instructed to perform a maximal vertical jump from a standing position with feet shoulder-width apartand hands on hips. No arm movement was allowed. A custom-built contact mat was used to record flight time (t; ms). Jump height (h) was computed using the following formula: h (m) = gt^2^/8, where g is the gravitational constant (9.81 m/s²) [61], and converted to cm. ICC = 0.94, CV% 5.17.

#### Stair climbing (SC) power test

Participants were instructed to climb a 6-step staircase as fast as possible, without missing one step and without using the handrail. The stair was located in a hallway of the university building and consisted of 6 steps of equal size, with 18 cm rise, 26.5 cm going and 4.5 cm nosing. Participants wore a sensor (DynaPort MoveTest, McRoberts, The Hague, NL) to determine the mean SC power for the rise phase of every step (see previous procedures [36]). The average power (SCP; W) over the six steps was calculated and used in further analyses. ICC = 0.90, CV% = 9.00 [39].

### Statistical analyses

Statistical analyses were performed with SPSS^®^ (version 29.0.1.0) and R-studio software (version 1.4.1564). The level of significance for all statistical tests was set at *p* < 0.05. Descriptive statistics were computed with age as a categorical variable. The physical characteristics and field test scores of four age groups are presented as mean ± standard deviation (SD) for males and females separately. Normality for the field test scores of the different groups was checked with Shapiro-Wilk (for samples < 50) or Kolmogorov-Smirnov tests (for samples > 50). Power and strength values in the HGS, STSP and SCP tests were normalized to body weight. To compare interindividual variability in performance across tests and age groups, coefficients of variation (CV, in %) were calculated as follows:

$$\:CV\left(\%\right)=\frac{SD\:age\:group}{mean\:age\:group}{\times}\:100$$

The relationship between age and test performance was assessed using segmented (piecewise) regression analysis. Age was treated as a continuous variable, and test scores were normalized relative to the mean of the youngest group (19–39 years). To note, time in the TUG was converted by the following formula:

$$\:Score\:\left(\%\right)=\frac{mean\:youngest\:group}{individual\:mean}\:{\times}\:100$$

This was done to allow comparison with other tests, so that the score in % of TUG decreased when duration of the test increased (i.e., worsening of performance). According to previous methods [62], an iterative approach was used to identify potential points at which a significant change in slope occurred. Various age points were evaluated (35, 40, 45, 50, 55, 60, 65, 70, and 75 years) within 30-year intervals (20–50, 25–55, 30–60, 35–65, 40–70, 45–75, 50–80, 55–85 and 60–90 years). The 30-year intervals were used to avoid that data far from a certain age point would contaminate the identification of a change in slope within a given age interval. The breakpoint was observed at 60 years in five out of six tests, with STSP showing no breakpoint in the regression models. The final model parameters of all tests included age, age60+ (if significant), sex (if significant), age-by-sex (if significant) and age60+-by-sex (if significant). To note, age represented the participant’s actual age, while age60 + denoted the number of years above 60. Concretely, age60 + equaled 0 for individuals aged 60 or younger, while age60 + equaled the difference between the participant’s age and 60 for individuals aged older than 60.

Differences in the relationship between age and test score across the six field tests were assessed. Linear mixed modeling, using the lmer function from the R-package lme4, was used to compare the slopes of the six regression models. The SCP regression model was used as reference because it had the steepest slope, i.e., the highest β coefficient for both age and age60 + in the regression model. 60 years was set as the age at which a significant change in slope occurred because this point was identified as the breakpoint in five out of six models. Performance score (in percentage) was included as dependent variable. Test (categorical variable), age, age60+, test-by-age and test-by-age60 + were entered as predictors in the model. Sex was not included in the model because neither the main effect of sex nor the sex-by-age(60+) and sex-by-test interaction effect were significant. Subject was treated as a random effect to correct for the repeated measures design. The comparison between tests was visualized by plotting the six distinct regressions into one graph.

To test the assumptions for (multilevel) regression models, we checked whether the models’ residuals were normally distributed, both by visual inspection of the Q-Q plots and histogram as well as by Kolmogorov-Smirnov tests. The residuals for all regression models were normally distributed (regression models per field test *p* > 0.05, multilevel regression model *p* > 0.01).

## Results

Table 1 summarizes physical characteristics and field test scores of the sample. Most field test scores showed a normal distribution within the different age and sex groups at *p* > 0.05. The following field tests were normally distributed at *p* > 0.01: STSP in men aged 40–54 years and TUG in women aged 40–54 years. The coefficient of variation appeared highest in the oldest age group, and lower in TUG, STSP and MGS compared to HGS, CMJ and SCP.

**Table 1:** Descriptive statistics (mean ± SD)

	19–39 years		40–54 years		55–64 years		≥ 65 years	
	♂ (*n* = 20)	♀ (*n* = 31)	♂ (*n* = 24)	♀ (*n* = 26)	♂ (*n* = 48)	♀ (*n* = 48)	♂ (*n* = 53)	♀ (*n* = 54)
**Physical characteristics**								
Age (years)	29.84 ± 5.23	26.59 ± 3.92	48.86 ± 4.39	49.30 ± 4.52	61.62 ± 2.69	59.26 ± 3.04	70.96 ± 4.56	71.54 ± 5.27
Weight (kg)	80.22 ± 14.54	67.43 ± 13.22	91.85 ± 9.83	69.61 ± 13.30	83.13 ± 13.05	66.82 ± 11.47	83.21 ± 9.98	65.94 ± 11.37
Height (m)	1.80 ± 0.07	1.66 ± 0.05	1.81 ± 0.09	1.67 ± 0.07	1.77 ± 0.06	1.65 ± 0.05	1.75 ± 0.06	1.61 ± 0.06
BMI (kg/m²)	24.56 ± 3.83	24.44 ± 4.92	28.02 ± 3.18	24.92 ± 4.38	26.53 ± 3.81	24.62 ± 4.23	27.24 ± 2.99	25.53 ± 4.36
**Field tests**								
HGS (kg/kg)	0.60 ± 0.10	0.47 ± 0.11	0.55 ± 0.11	0.42 ± 0.07	0.55 ± 0.1	0.45 ± 0.08	0.51 ± 0.1	0.41 ± 0.1
CV%	15.8	23.5	19.4	16.0	19.2	18.5	19.0	24.5
CMJ height (cm)	31.43 ± 5.16	22.00 ± 5.01	26.87 ± 4.93	17.65 ± 3.88	23.17 ± 4.97	17.19 ± 3.63	18.83 ± 4.24	12.91 ± 3.98
CV%	16.4	22.8	18.3	22.0	21.5	21.1	22.5	30.8
STSP (W/kg)	5.49 ± 0.81	4.01 ± 0.50	5.03 ± 0.76	3.82 ± 0.42	4.54 ± 0.66	3.57 ± 0.42	4.27 ± 0.6	3.2 ± 0.61
CV%	14.7	12.4	15.0	10.9	16.2	11.9	14.0	18.9
SCP (W/kg)	15.21 ± 3.18	11.89 ± 2.38	12.13 ± 1.66	9.57 ± 1.78	10.54 ± 1.7	8.7 ± 1.76	8.2 ± 2.15	6.17 ± 1.98
CV%	20.9	20.0	13.7	18.6	14.6	20.3	26.2	32.1
MGS (m/s)	2.45 ± 0.31	2.15 ± 0.25	2.25 ± 0.26	2.07 ± 0.18	2.27 ± 0.37	2.04 ± 0.31	2.07 ± 0.39	1.84 ± 0.32
CV%	12.8	11.4	11.6	8.5	16.4	15.2	18.9	17.5
TUG (s)	4.72 ± 0.34	5.16 ± 0.58	4.97 ± 0.43	5.42 ± 0.65	5.72 ± 0.78	5.55 ± 0.59	6.03 ± 0.87	6.61 ± 1.14
CV%	7.2	11.3	8.7	12.0	13.6	10.7	14.4	17.2

HGS = handgrip strength; CMJ = countermovement jump; CV = coefficient of variation, representing inter-individual variability in the performance scores in each age group; STSP = sit-to-stand power; SCP = stair-climbing power; MGS = maximal gait speed; TUG = timed-up-and-go

**Fig. 1:**
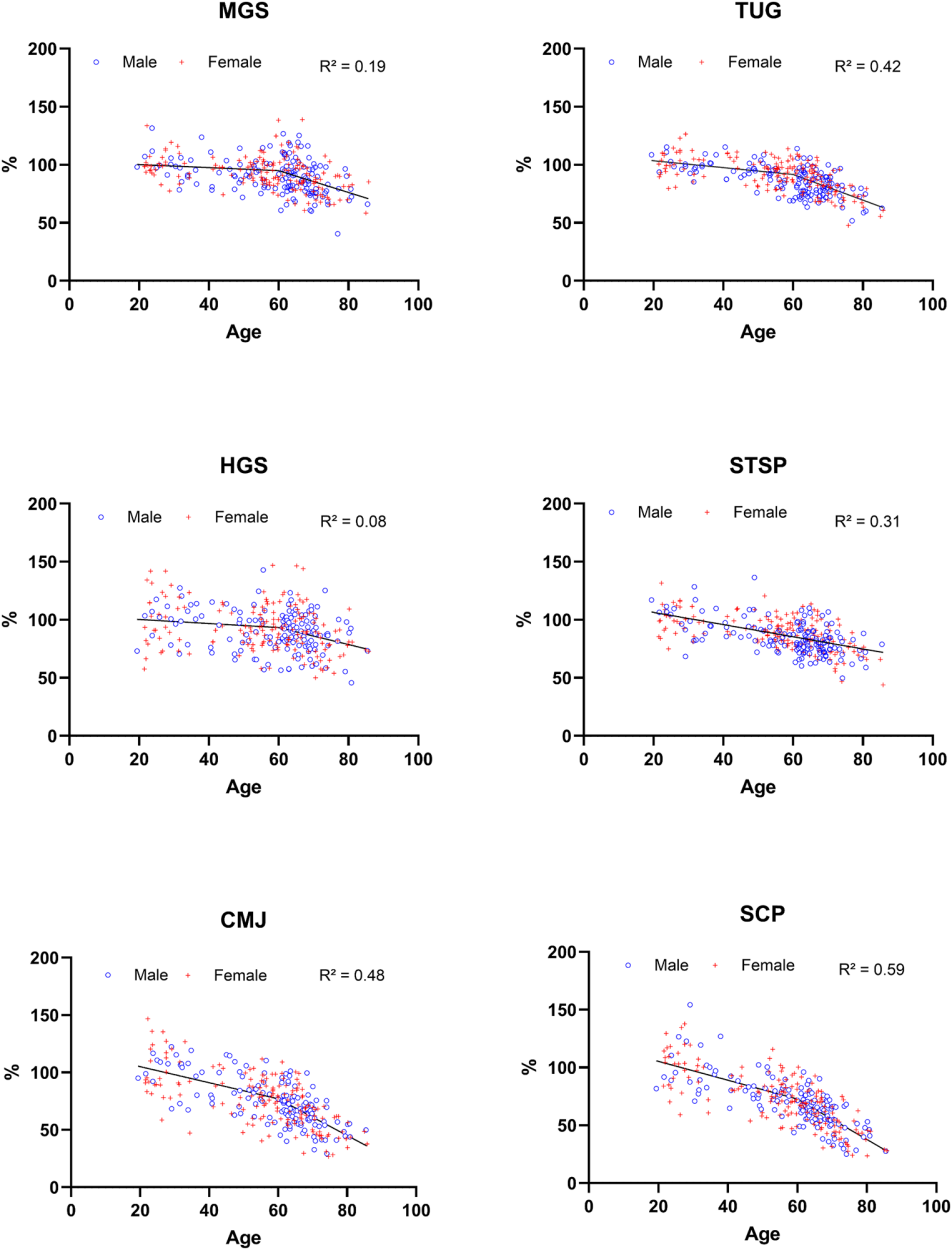
Visual representation of piecewise regression models for all field tests. HGS = handgrip strength; CMJ = countermovement jump; STSP = sit-to-stand power; SCP = stair-climbing power; MGS = maximal gait speed; TUG = timed-up-and-go; R^2^ = coefficient of determination. Blue circles represent males, red crosses females

Table 2; Fig. 1 illustrate the regression models for the six field tests. Across all tests, the β-coefficients indicate a negative relationship between age and test performance (R² = 0.08–0.59). In all tests, except the STSP, the decline in performance with increasing age was significantly steeper after age 60 compared to before. Before age 60, the decline was significant for the SCP, CMJ, STSP and TUG tests (all *p* < 0.05), but not for the HGS and MGS tests (*p* > 0.05). After the age of 60, the decline was significant for all tests (all *p* < 0.05). Significant age-by-sex and age-60+-by-sex interactions (*p* < 0.05) were only observed in the TUG test, with males showing a greater decline before the age of 60 and females showing a greater decline after the age of 60.

**Table 2:** Results of the piecewise regression analysis for all field tests

Variable	Intercept	Age	Sex	Age 60+	Age-by-sex	Age 60+-by-sex	*R*²
**HGS**							0.08
β	100.38^***^	-0.17^n.s^.		-0.56^*^			
CI	[94.23; 106.53]	[-0.37; 0.02]		[-1.08; -0.04]			
SE	3.13	0.10		0.27			
**CMJ**							0.48
β	105.53^***^	-0.70^***^		-0.91^***^			
CI	[100.13; 110.92]	[-0.87; -0.53]		[-1.37; -0.45]			
SE	2.74	0.09		0.23			
**STSP**							0.31
β	106.68^***^	-0.52^***^					
CI	[103.13; 110.24]	[-0.61; -0.44]					
SE	1.81	0.04					
**SCP**							0.59
β	105.83^***^	-0.81^***^		-0.94^***^			
CI	[100.97; 110.70]	[-0.97; -0.66]		[-1.35; -0.53]			
SE	2.47	0.08		0.21			
**MGS**							0.19
β	100.17^***^	-0.13^n.s^.		-0.83^***^			
CI	[95.65; 104.69]	[-0.27; 0.02]		[-1.21; -0.45]			
SE	2.30	0.07		0.20			
**TUG**							0.44
β	105.67^***^	-0.45^***^	-3.31^n.s^.	-0.35^n.s^.	0.27^*^	-0.81^**^	
CI	[99.96; 111.39]	[-0.62; -0.27]	[-10.45; 3.82]	[-0.80; 0.10]	[0.04; 0.49]	[-1.41; -0.22]	
SE	2.91	0.09	3.63	0.23	0.11	0.30	

HGS = handgrip strength; CMJ = countermovement jump; STSP = sit-to-stand power; SCP = stair-climbing power; MGS = maximal gait speed; TUG = timed-up-and-go; β = regression coefficient; CI = confidence interval; SE = standard error; R^2^ = coefficient of determination; Age = age (years); Age 60 + = age (years) – 60 (for age >60) or 0 (for age ≤60); Sex: male = 0, female = 1; ^***^p < 0.001; ^**^p = 0.01; ^*^p = 0.05; n.s. = *p* > 0.05

**Fig. 2:**
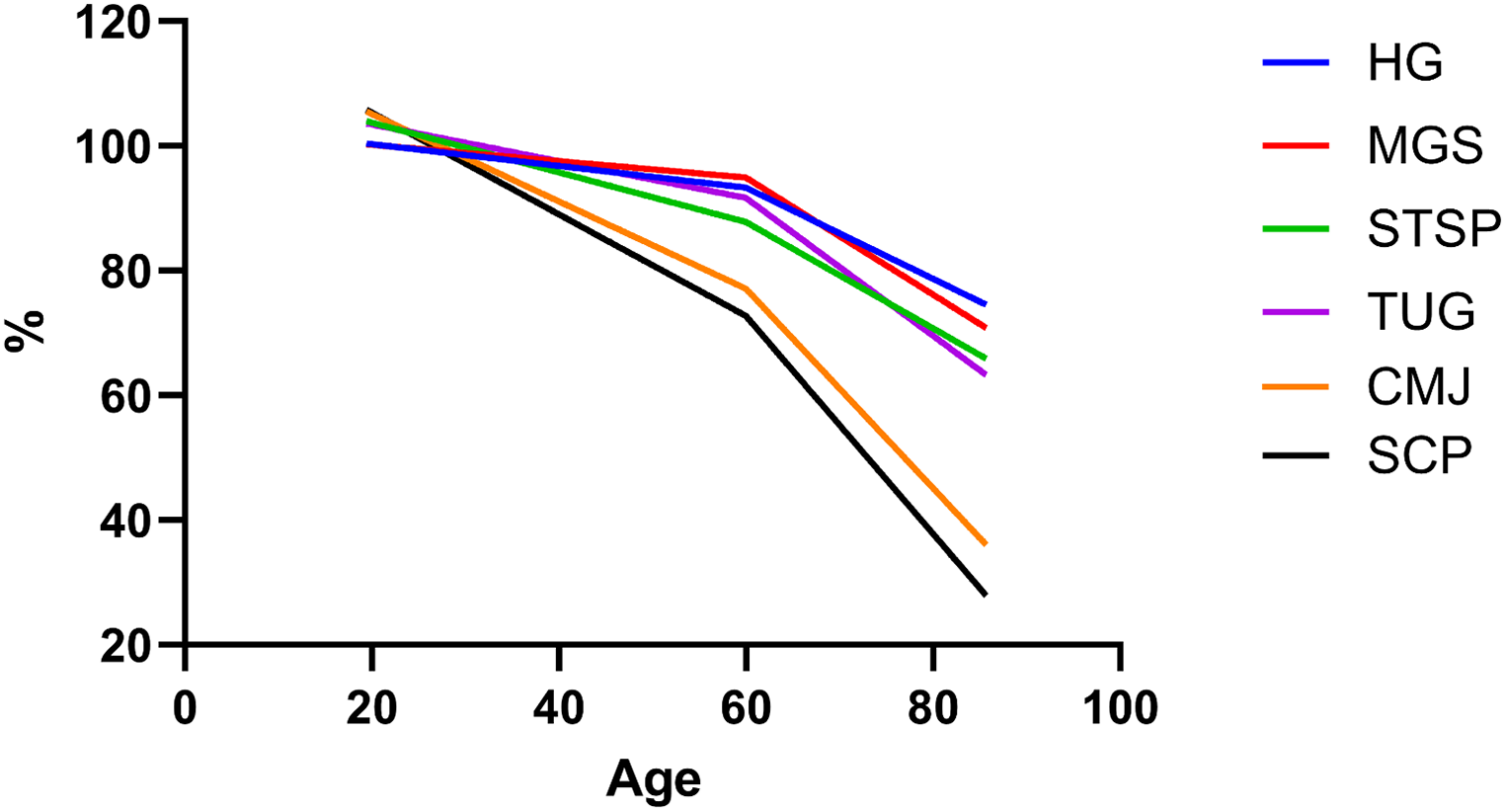
Visual representation of comparison of age-related declines between field tests. HGS = handgrip strength; MGS = maximal gait speed; STSP = sit-to-stand power; TUG = timed-up-and-go; CMJ = countermovement jump; SCP = stair-climbing power. The SCP served as the reference for comparison between tests. Before age 60, the rate of decline in CMJ was not significantly different from the SCP, while all other tests showed a less steep decline compared to SCP. At age 60, the acceleration in decline rates for all tests, except STSP, was not statistically different from the SCP

Table 3; Fig. 2 present the comparison between tests. In Table 2, the highest β coefficient for both age and age60 + was reported in the SCP. Therefore, SCP was used as a reference for comparison of age-related declines between tests. Before the age of 60, age-related declines were significantly lower in HGS, STSP, MGS and TUG compared to SCP (*p* < 0.001). This difference in the rate of decline compared to SCP increased even further after the age of 60 for the STSP (i.e., an additional difference in the rate of decline of 0.5% per year, *p* = 0.031), but not for the HGS, MGS and TUG (all *p* > 0.05). No differences in the rates of decline where found in CMJ compared to SCP (*p* > 0.05 both before and after 60 years).

**Table 3:** Results of linear mixed model analysis comparing field tests

	β (CI)	*p*-value
**Intercept**	105.83 (101.04; 110.62)	*p* < 0.001
**Age**	-0.81 (-0.97; -0.66)	*p* < 0.001
**Age 60+**	-0.94 (-1.35; -0.53)	*p* < 0.001
**Test**		
HGS	-5.45 (-10.83; -0.07)	0.047
CMJ	-0.31 (-5.69; 5.08)	0.911
STSP	-1.73 (-7.11; 3.66)	0.530
MGS	-5.66 (-11.05; -0.28)	0.039
TUG	-2.20 (-7.58; 3.66)	0.424
**Age-by-test**		
HGS	0.64 (0.47; 0.81)	*p* < 0.001
CMJ	0.12 (-0.06; 0.29)	0.185
STSP	0.41 (0.24; 0.58)	*p* < 0.001
MGS	0.69 (0.52; 0.86)	*p* < 0.001
TUG	0.52 (0.35; 0.69)	*p* < 0.001
**Age 60+-by-test**		
HGS	0.38 (-0.08; 0.84)	0.105
CMJ	0.03 (-0.43; 0.49)	0.895
STSP	0.50 (0.05; 0.96)	0.031
MGS	0.11 (-0.35; 0.57)	0.640
TUG	0.12 (-0.33; 0.58)	0.598

HGS = handgrip strength; CMJ = countermovement jump; STSP = sit-to-stand power; SCP = stair-climbing power; MGS = maximal gait speed; TUG = timed-up-and-go; β = regression coefficient; CI = confidence interval; Age = age (years); Age 60 + = age (years) – 60 (for age >60) or 0 (for age ≤60)

## Discussion

The current study investigated the ability of various field tests to detect age-related differences in physical and muscle function across the adult lifespan. As expected, tests that required high lower-limb muscle power, like the CMJ and SCP, were better at detecting early age-related differences compared to the HGS, MGS, TUG and STSP. Specifically, the annual decline rates ranged from − 0.70 to -0.81% before age 60, and these rates increased to between − 1.61 and − 1.75% after age 60. Our cross-sectional findings align with the decline rates in knee-extensor muscle power reported in a previous 10-year follow-up study in 489 adults aged 19–68 years at baseline. This longitudinal study found annual declines of -1.1 to -1.4% per year in middle-aged adults (40–60 years) and − 2.2 to 2.4% in individuals aged 60 or older [14]. Similar decline rates were found for leg-extensor power assessed by the Nottingham power rig, with the annual rate of decline between − 1.3% to -1.7% in middle-aged adults (40–60 years) and between − 1.7% to -2.3% in older adults (60–80 years) [62].

As mentioned in the introduction section, multiple underlying mechanisms can be proposed to explain these substantive declines in muscle power with ageing. In addition to the age-related shift from fast-to-slow muscle fiber phenotype [16], a major determinant of the ability to produce force rapidly is the increase of muscle activation at the onset of contraction [63], which significantly diminished with ageing [21]. Given that CMJ and SCP involve stretch-shortening cycle muscle actions, impaired utilization of elastic energy due to neural and structural changes in aged muscles and tendons may also contribute to the decline in muscle power [64, 65].

Age was not a good predictor of HGS across the adult lifespan, as demonstrated by a low coefficient of determination, primarily because of the non-significant relationship between age and HGS before 60 years. The emphasis on upper-limb rather than lower-limb strength may result in reduced responsiveness of HGS in detecting early age-related [31, 32] and training-induced [66] adaptations. A different contributing factor could be the focus of the HGS test on maximal muscle strength rather than maximal muscle power, which typically demonstrates less pronounced age-related declines [14, 57].

The lower ability of the MGS, TUG and STSP to detect age-related differences compared to the SCP during early life stages may be attributed to the less challenging nature of the tests. Previous research underscores the importance of using measurement tools with minimal floor and ceiling effects for the intended purpose and population [67]. In our study, we posit that the three tests were not optimally suited for our population due to the imposed restrictions that hindered participants from performing at their full capacity. Hindering participants to perform at their full capacity may result in less variability in performance scores between young individuals, consequently leading to less pronounced age-related differences. Although performance variables did follow the normal distribution in all age groups, our results seem to support the notion of less variability in the younger age groups for MGS, TUG and STSP compared to SCP and CMJ (see CV% in Table 1), which is at least suggestive of a ceiling effect.

From the age of 60 onwards, there was a similar change in the rate of decline in the CMJ, HGS, MGS and TUG when compared to SCP. Given that the CMJ and SCP exhibited similar rates of decline before 60, the comparable changes in slope after 60 imply that the total age-related decreases in the two tests have similar magnitudes. Conversely, HGS, MGS and TUG had lower rates of decline compared to SCP before the age of 60, indicating that the overall age-related decline after 60 years old still remained smaller compared to SCP. More specifically, the overall age-related decline after 60 years was − 1.75%/year for SCP in comparison to -0.73%/year for HGS, -0.96%/year for MGS, and − 0.80% to -1.34%/year for TUG. Notably, as illustrated by the slopes of the tests after the age of 60 in Fig. 2, the STSP stood out as the only test exhibiting no significant acceleration in the rate of decline at 60 years old. This means that the difference in the rate of decline between STSP and SCP prior to the age of 60 years increased even further after the age of 60 years.

Previous reports have attempted to pinpoint the breakpoint at which age-related declines in performance accelerate. In a review paper of Ferruci et al. (2016), longitudinal data on gait speed in individuals across all life stages were analyzed from the InCHIANTI study. They reported that the rate of decline in maximal gait speed accelerated between the age of 60–70 years, which is very similar to our findings [56]. Likewise, the Copenhagen Sarcopenia Study, which examined a Danish cohort aged 20–93 years, observed that maximal gait speed significantly deviated from the young reference values starting in the age category of 60–69 years [57]. A cross-sectional study of Samson et al. in 74 healthy women and 81 healthy men (age range of 20–90 years) found a breakpoint in the age-related decline in HGS at age 55 in women, but no breakpoint for HGS in men, nor for TUG in either sex [53]. However, upon visual inspection, the data in their scatterplots do seem to indicate that average TUG values are quite similar between 20 and 50 years of age. The limited sample size per sex in the study of Samson et al. may have been insufficient to detect a breakpoint [53]. Data from Vianna et al. in a large cross-sectional sample (1.787 men (18–91 years) and 861 women (18–88 years)) show a faster decline in HGS at 30 years in men and 50 years in women [52]. To compare our decline rates for HGS in middle-aged individuals (i.e., early decline) with those reported by Vienna et al., we calculated the HGS values for a 30- and 50-year old man and women with average height (♂ 170 cm, ♀ 160 cm) and weight (♂ 82 kg, ♀ 65 kg). According to Vianna et al., men would exhibit ~ 13.0% lower HGS at age 50 compared to 30, while women would show ~ 5.9% lower values at age 50 compared to 30 [52]. This decline rate is higher for men and lower for women compared to our current study, which indicated that a 50-year old individual would exhibit 8.1% lower values than the average value of the young reference sample (20–40 years). However, our decline rate in HGS is very similar to the findings of the Copenhagen Sarcopenia Study, which reported 7.2% lower values in men aged 50–59 years compared to those aged 30–39 years, and 8.6% lower values in women [57].

Several limitations should be considered when interpreting the current study’s findings. Firstly, the cross-sectional study design illustrates age-related differences at population level, but does not provide information about individual changes over time. Secondly, the inclusion criteria focusing on well-functioning community-dwelling adults may limit the generalizability of the results to broader populations. Finally, researchers and clinicians use various protocols and measuring techniques to assess performance on physical and muscle function tests. For example, in SC and STS tests, different methodologies exist to estimate the power production, including wearable sensor-based assessments and non-instrumented versions using test duration to calculate power [35, 38, 39, 43, 68, 69]. As both methodologies can differentiate between age groups [36, 38, 43, 54, 55], it might be questioned what the added value is of a sensor-based approach over a more simple, feasible method in which power is estimated based on total stair ascent or STS duration. In the case of stair ascent, a duration-based formula can only estimate the minimal power necessary to lift the body from one step to the other in a certain amount of time. When a participant is able to run during stair ascent, the vertical power experienced by of the body center of mass will overshoot this minimal power. This makes the sensor-based approach more appropriate in well-functioning adults, as supported by our previous work [38]. In the case of STS, a sensor or smartphone can provide additional information on the movement strategies and movement phases during the test [35, 70], which allows for a more accurate detection of the concentric power-producing phase. However, we do acknowledge that further optimization and simplification of the instrumented methodologies in both SCP and STSP is necessary before it can be implemented in clinical or home-based settings. Another aspect that needs consideration in stair-climbing assessments, is the staircase that is used, in particular with regard to the number of steps [68, 71]. To investigate the potential differential results of the number of steps, we previously compared power production on a 3-, 6- and 12-step staircase in well-functioning older adults. While mean power production was higher with increasing number of steps, very high correlation coefficients were found between the different staircases (*r* = 0.85–0.95). This indicates that, although absolute values differ, it probably does not matter which type of staircase is used, as long as clinicians consistently use the same stair model when comparing individual performances [38]. Another difference in test protocols applies to the STS test. Roughly two main test approaches are used to evaluate performance: recording either the maximum repetitions within a set time [72] or the time taken to complete a specific number of repetitions [73]. In addition, the CMJ jump height can be determined using different calculation methods [74]. These discrepancies should be considered before extending the findings to comparable but different tests.

## Conclusions

Beyond the age of 60, age-related declines in physical capabilities can be effectively identified through various commonly used field tests. However, for younger, middle-aged individuals, detecting functional decline may necessitate a different approach, focusing on field tests that specifically target lower-limb power and challenge individuals at a higher end of the performance spectrum. The SCP and CMJ stand out as responsive tests for identifying age-related declines across the entire adult lifespan. Whether these early changes in SCP and CMJ are predictive of future negative health outcomes remains to be investigated in longitudinal study designs.

## Data Availability

The data that support the findings of this study are available upon request to the corresponding author. The data are not publicly available due to privacy or ethical restrictions.
